# COVID-19 vaccination and the risk of autoimmune diseases: a Mendelian randomization study

**DOI:** 10.3389/fpubh.2024.1322140

**Published:** 2024-03-14

**Authors:** Jiayi Shan, Xiaoyun Hu, Tianzhu Chen, Yuyang Wang, Baoyi Huang, Yijun Xin, Hua Xu

**Affiliations:** ^1^The First Clinical Medical School, Guangzhou University of Chinese Medicine, Guangzhou, China; ^2^Department of Pediatrics, The First Affiliated Hospital of Guangzhou University of Chinese Medicine, Guangzhou, China

**Keywords:** COVID-19 vaccination, autoimmune diseases, Mendelian randomization study, multiple sclerosis, ulcerative colitis, immunity, vaccination, COVID-19

## Abstract

**Background:**

In recent times, reports have emerged suggesting that a variety of autoimmune disorders may arise after the coronavirus disease 2019 (COVID-19) vaccination. However, causality and underlying mechanisms remain unclear.

**Methods:**

We collected summary statistics of COVID-19 vaccination and 31 autoimmune diseases from genome-wide association studies (GWAS) as exposure and outcome, respectively. Random-effects inverse variance weighting (IVW), MR Egger, weighted median, simple mode, and weighted mode were used as analytical methods through Mendelian randomization (MR), and heterogeneity and sensitivity analysis were performed.

**Results:**

We selected 72 instrumental variables for exposure (*p* < 5 × 10^−6^; r2 < 0.001, genetic distance = 10,000 kb), and MR analyses showed that COVID-19 vaccination was causally associated with an increased risk of multiple sclerosis (MS) (IVW, OR: 1.53, 95% CI: 1.065–2.197, *p* = 0.026) and ulcerative colitis (UC) (IVW, OR: 1.00, 95% CI: 1.000–1.003, *p* = 0.039). If exposure was refined (*p* < 5 × 10^−8^; r2 < 0.001, genetic distance = 10,000 kb), the associations became negative. No causality was found for the remaining outcomes. These results were robust to sensitivity and heterogeneity analyses.

**Conclusion:**

Our study provided potential evidence for the impact of COVID-19 vaccination on the risk of MS and UC occurrence, but it lacks sufficient robustness, which could provide a new idea for public health policy.

## Background

COVID-19 is a rapidly spread global disease caused by Severe Acute Respiratory Syndrome Coronavirus 2 (SARS-CoV-2) since the first outbreak in December 2019 and was declared a global pandemic in March 2020 by the World Health Organization (1). Despite quarantine measures, the incidence and mortality of COVID-19 still increased exponentially and continuously, with 769 million confirmed cases and 6.9 million deaths reported globally as of August 9, 2023 (2), which made it appear that COVID-19 vaccination was a very critical healthcare intervention. So far (November 2022), there have been six vaccines granted marketing approval by the European Medicines Agency (EMA), including two RNA vaccines (Pfizer-BioNTech and Moderna), two adenovirus vaccines (AstraZeneca and Janssen), a recombinant adjuvanted vaccine (Novavax), and one inactivated adjuvanted vaccine (COVID-19 vaccine Valneva) (3). However, many vaccine-related side effects and complications have been reported (4–6), a large proportion of which were autoimmune diseases (ADs). It has been hypothesized that this is probably due to cross-reactivity between the SARS-Cov-2 proteins and human proteins (7).

Severe complications of COVID-19 vaccines were reported incorporating thrombotic thrombocytopenia (8), Vaccine-induced immune thrombotic thrombocytopenia (9) (VITT), Immune thrombocytopenia (ITP) (9), myocarditis or pericarditis (10), Guillain-Barre syndrome (GBS) (11), Bell’s palsy (12), neuromyelitis optica spectrum disorder (NMOSD) (13) and multiple sclerosis (MS) (14) and so on. These possible adverse reactions increase vaccine hesitancy, especially for special populations (15). Therefore a deeper insight into the causality and magnitude of the effect of the COVID-19 vaccine on its complications may be beneficial for the identification of at-risk patients and design of preventive or therapeutic interventions. However, observational studies are susceptible to other factors such as unclear underlying undetected ADs in patients. Whether COVID-19 vaccination causally increases ADs remains unknown, which may relate to the safety of the COVID-19 vaccine.

In this study, we conducted a two-sample Mendelian randomization (MR) study to assess the association between predisposition to COVID-19 vaccine and major ADs. Since MR is a method of integrating pooled data from genome-wide association studies (GWAS), similar to a randomized controlled trial, using genetic variation as an instrumental variable (IV), it is generally less likely to be affected by residual confounders and reverse causality, thus strengthening the causal relationship between exposure and outcome (16). Ethical approval was not applicable because the summary statistics used are publicly available.

## Method

### Study design

**Figure 1:**
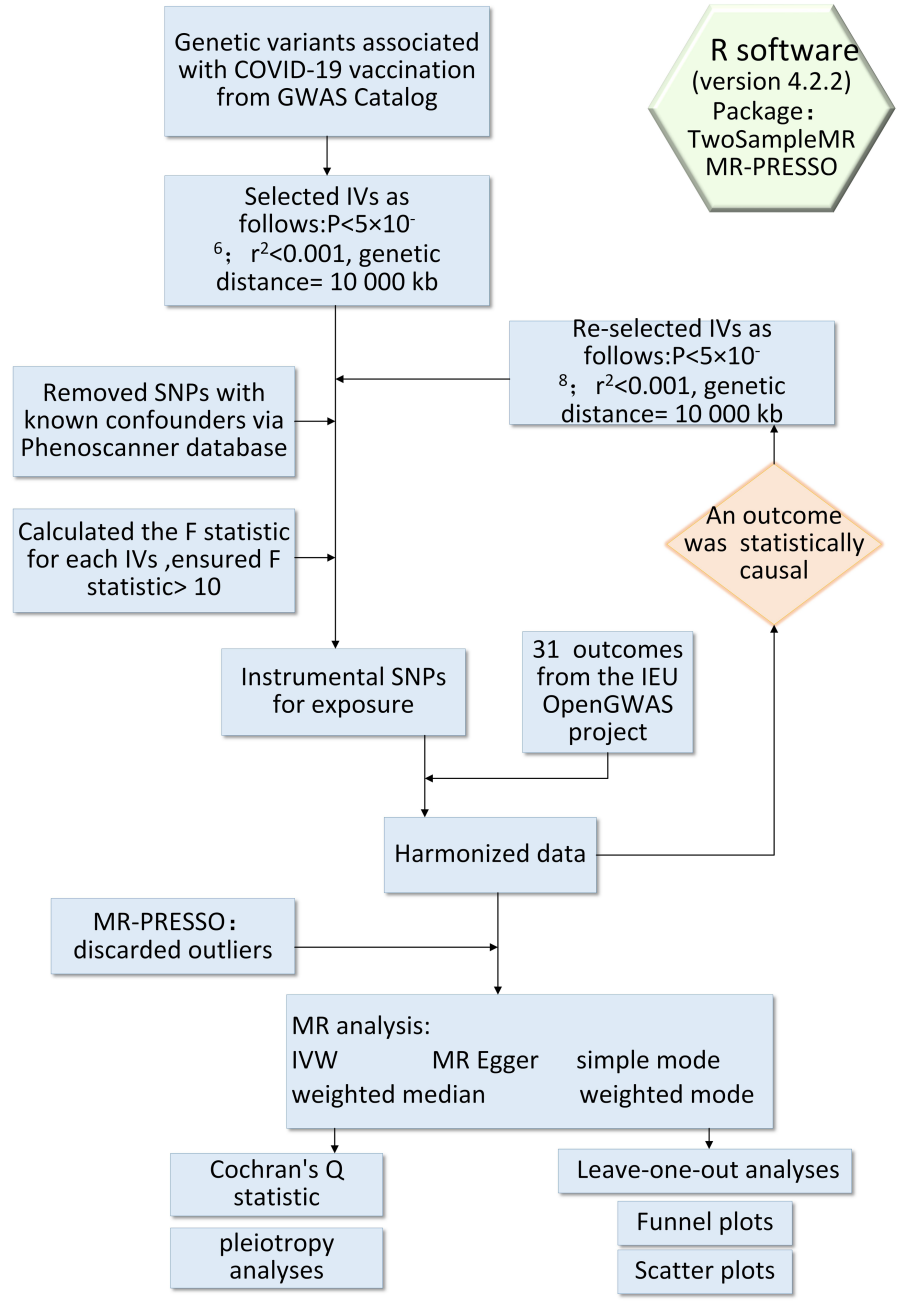
Flowchart of this study.

In this study, we selected Single Nucleotide Polymorphism (SNP) as IV from the GWAS dataset to explore the causal link between exposure and outcome. This study met three critical assumptions of the two-sample MR design: (1) all selected IVs were strongly associated with exposure; (2) all selected IVs were not associated with confounders between exposure and outcome; (3) all selected IVs affected outcome exclusively via exposure without affecting other pathways (17). The overall flow of work for our analysis was summarized in Figure 1.

### Data source

For MR analysis, COVID-19 vaccination as the exposure was obtained from publicly available GWAS Catalog summary (18) results (COVID-19 vaccination, 3,055,558 cases and 136,947 controls). All cases and controls were of European ancestry (19). They have chosen an age range of 30–80 years for the study because by October 31, 2021, everyone in this age group would be eligible to receive the first dose of COVID-19 for at least 4 months.

31 ADs were obtained as outcomes from the IEU OpenGWAS project (20), in which the 31 ADs included have been previously reported in the literature and were categorized into 7 groups including vascular disease, nervous and mental diseases, rheumatic disease, dermopathy, nephropathy, digestive diseases, and others. All cases and controls in these studies were European. In addition, there was no significant overlap between the populations of the GWAS studies. Detailed information can be found in Table 1.

**Table 1:** Characteristics of the COVID-19 vaccination and 31 disease GWAS cohorts.

Phenotype_name	GWAS_ID	N_case	N_control	Sample size
COVID-19 vaccination	GCST90255613	3,055,558	136,947	3,192,505
**• Vascular and cardiac disease**				
Idiopathic thrombocytopenic purpura	finn-b-D3_ITP	394	216,099	216,493
Other nonthrombocytopenic purpura	finn-b-D3_OTHNONTHROMBOCYTOPENPURPURA	86	216,099	216,185
Secondary thrombocytopenia	finn-b-D3_SCNDTHROMBOCYTOPENIA	94	216,099	216,193
Thrombocytopenia, unspecified	finn-b-D3_THROMBOCYTOPENIANAS	854	216,099	216,953
Pericarditis	finn-b-I9_PERICARD	484	156,711	157,195
Myocarditis	finn-b-I9_MYOCARD	829	116,926	117,755
**• Nervous and mental diseases**				
Generalized epilepsy	finn-b-GE	1,781	212,532	214,313
Guillain-Barre syndrome	finn-b-G6_GUILBAR	213	215,718	215,931
**• Rheumatic disease**				
Psoriatic arthropathies	finn-b-M13_PSORIARTH_ICD10	1,455	217,337	218,792
Ankylosing spondylitis	ukb-a-88	968	336,191	337,159
Rheumatoid arthritis	finn-b-M13_RHEUMA	6,236	147,221	153,457
Systemic lupus erythematosus	ebi-a-GCST003156	5,201	9,066	14,267
Multiple Sclerosis	finn-b-G6_MS	1,048	217,141	218,189
Kawasaki disease	ebi-a-GCST90014243	119	6,071	6,190
**• Dermopathy**				
Psoriasis	finn-b-L12_PSORIASIS	4,510	212,242	216,752
Urticaria	finn-b-L12_URTICARIA	5,066	212,464	217,530
Hidradenitis suppurativa	finn-b-L12_HIDRADENITISSUP	409	211,139	211,548
Lichen planus	finn-b-L12_LICHENPLANUS	1,865	212,242	214,107
Vasculitis limited to skin	finn-b-L12_VASCULITISSKIN	288	207,482	207,770
**• Nephropathy**				
Nephrotic syndrome	finn-b-N14_NEPHROTICSYND	480	214,619	215,099
IgA nephropathy	ieu-a-1081	977	4,980	5,957
Membranous nephropathy	ebi-a-GCST010005	2,150	5,829	7,979
**• Digestive diseases**				
Crohns disease	ukb-a-103	1,032	336,127	337,159
Ulcerative colitis	ukb-b-7584	2,439	460,494	462,933
Celiac disease	ebi-a-GCST005523	11,812	229	23,649
Irritable bowel syndrome	finn-b-K11_IBS	4,605	182,423	187,028
Primary biliary cirrhosis	ebi-a-GCST005581	2,861	8,514	11,375
Primary sclerosing cholangitis	ieu-a-1112	2,871	12,019	14,890
**• Other**				
Type 1 diabetes	finn-b-T1D_STRICT	2,542	182,573	185,115
Other autoimmune Haemolytic anaemias	finn-b-D3_AIHA_OTHER	127	218,396	218,523

### Genetic instrument selection

(1) In order to fulfill the first MR assumption for identification, the SNP must be strongly associated with the exposure variable (COVID-19 vaccination), however, to obtain a larger number of SNPs as IVs, we first selected a relatively loose threshold of statistical significance (*p* < 5 × 10^−6^; r^2^ < 0.001, genetic distance = 10,000 kb) (21). If the exposure was found to be statistically causal for an outcome, the eligibility criteria for exposure could be appropriately changed to (*p* < 5 × 10^−8^; r^2^ < 0.001, genetic distance = 10,000 kb), and analyzed again more precisely with the specific disease. (2) For the second assumption of MR, a query was performed in the Phenoscanner (22) database to determine that the included SNPs were not associated with known confounders, and SNPs with potential bias were removed. (3) Finally, we calculated the F statistic for the IVs to assess the extent of weak instrumental bias. It was ensured that the retained IVs had F statistic>10 to minimize bias caused by weak instrumental variables. The formula for calculating the F statistic is as follows (23):


F=R^2/1−R^2∗n−k−1/kR^2=2∗MAF∗1−MAF∗Beta^2

### Statistical analysis

We used “TwoSampleMR” and “MR-PRESSO” packages in R software (version 4.2.2) to perform a two-sample MR Analysis for COVID-19 vaccination and 31 ADs. Random-effects inverse variance weighting (IVW), MR Egger, weighted median, simple mode, and weighted mode were used for analysis, and IVW was used as the primary method because it can provide relatively stable and accurate causal estimates by combining Wald estimates of each IV through a meta-analysis approach (23). In the summary of IVW results, only *p*-values<0.05 were used to estimate causal effects, and if the odds ratio (OR) > 1, the exposure is considered a risk factor for the outcome; otherwise, exposure is a protective factor (17). In order to present the results of the different MR methods, we constructed scatter plots using the TwoSampleMR package.

To avoid IVs acting on the results through pathways beyond exposure and to reduce the bias caused by horizontal pleiotropy, we use MR-PRESSO to detect broad horizontal pleiotropy in all results, and outliers identified will be discarded with the MR analysis being re-executed (24). To evaluate the robustness of the results, we performed MR-IVW and MR-Egger analyses using Cochran’s Q statistic for statistically significant results, testing for heterogeneity, which indicated no heterogeneity when *p*-values >0.05 (25). Funnel plots and leave-one-out analyses are also used to test heterogeneity, while symmetric funnel plots and leave-one-out analyses with no significant outliers are expected. Finally, we used the MR-egger method to assess the magnitude of pleiotropy via an intercept test, and when *p*-values >0.05, which indicates a weak pleiotropy, its effect was ignored (26).

## Result

### Selection of instrumental variables

A total of 1,175 SNPs were identified from our GWAS dataset as IVs (*p* < 5 × 10–6) for the COVID-19 vaccination, followed by clumping leaving 72 strong instrumental variants for further analysis (F statistics>10), details were seen in Supplementary Table S1. However, there were 7 SNPs were identified from our GWAS dataset as IVs (*p* < 5 × 10^−8^) for the COVID-19 vaccination after clumping, as seen in Supplementary Table S2. For more, we went through the above IVs one by one from the Phenoscanner, eliminating 7 SNPs with potential confounders (rs138896727, rs3748655, rs35267052, rs356991, rs145071856, rs113560707, rs116887540), seen in Table 2.

**Table 2:** Excluded SNPs from phenoscanner.

SNP	Confounding trait
rs138896727	Cause of death: peripheral vascular disease, unspecified
rs3748655	Platelet distribution width
rs35267052	Platelet count
rs356991	Red blood cell count
rs145071856	Self-reported gastroenteritis or dysentry
rs113560707	Cause of death: cholangitis
rs116887540	Diabetes diagnosed by doctor

As for the 33 outcomes, we used the “extract_outcome_data” function in the “TwoSampleMR” package to extract them directly from the IEU Open GWAS project and proceed to the next step of harmonization, during which palindromic SNPs were excluded (details in Supplementary Table S3). In the harmonization, celiac disease and primary biliary cirrhosis had no harmonized results because they did not have the same IVs extracted from COVID-19 vaccination. Subsequent to harmonization, the MR- presso global test showed that mean platelet volume (*p* < 0.001) indicated horizontal pleiotropy and the distortion test had an outlier value as rs67600240, whereas the rest of the MR analyses of COVID-19 vaccination with ADs showed no horizontal pleiotropy, details were seen in Supplementary Table S3.

### Mendelian randomization analysis

**Figure 2:**
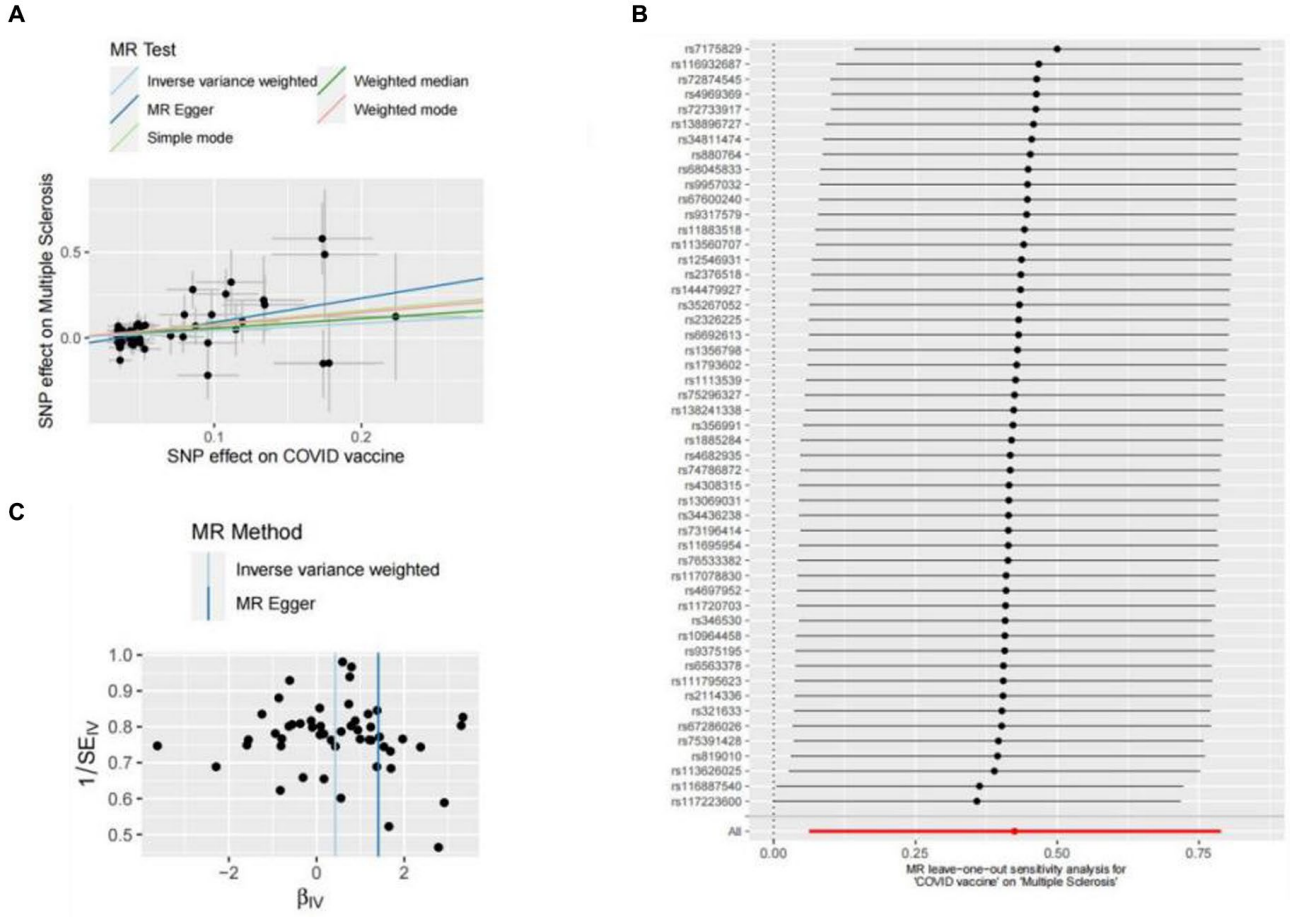
Scatter plots of the MR analyses for the association of COVID-19 vaccination and the risk of multiple sclerosis. **(A)** Causal effect of COVID-19 vaccination on multiple sclerosis; **(B)** Leave-one-out analyses for the causal estimates of COVID-19 vaccination on the risk of multiple sclerosis; **(C)** Funnel plot from COVID-19 vaccination on the risk of multiple sclerosis.

**Figure 3:**
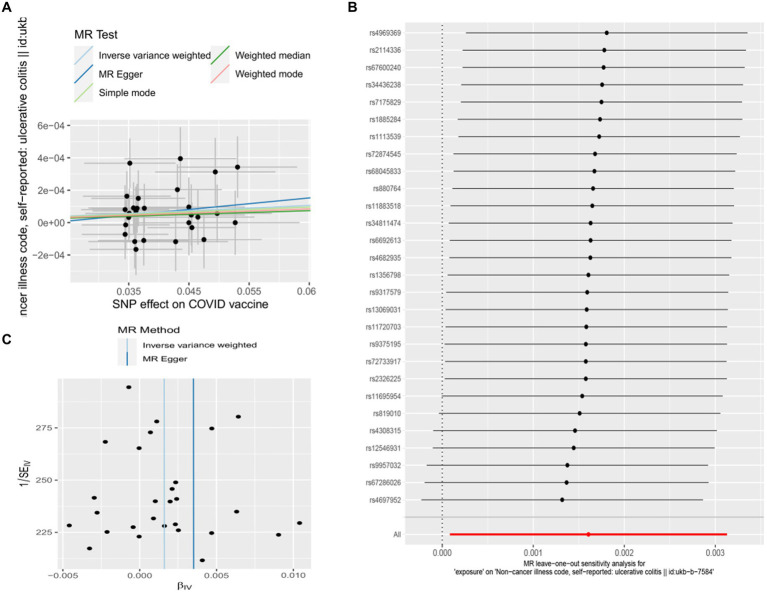
Scatter plots of the MR analyses for the association of COVID-19 vaccination and the risk of ulcerative colitis. **(A)** Causal effect of COVID-19 vaccination on ulcerative colitis; **(B)** Leave-one-out analyses for the causal estimates of COVID-19 vaccination on the risk of ulcerative colitis; **(C)** Funnel plot from COVID-19 vaccination on the risk of ulcerative colitis.

Genetic liability to most ADs were not significantly associated with COVID-19 vaccination when we chose a threshold of *p* < 5 × 10^−6^ for the instrumental variables in 5 primary MR methods. However, we found there were significant associations between vaccination and increased multiple sclerosis (MS) (IVW, OR: 1.53, 95% CI: 1.065–2.197, *p* = 0.026) and the estimates were generally similar using MR-Egger and the weighted median methods, shown in the scatter plot (Table 3; Figure 2A). Secondly, there was a statistically significant causal relationship between ulcerative colitis (UC) and COVID-19 vaccination (IVW, OR: 1.00, 95% CI: 1.000–1.003, *p* = 0.039). Except for the Inverse variance weighted method, which was significantly different, the remaining four methods did not show any statistically different (Table 3; Figure 3A).

**Table 3:** Associations of multiple sclerosis, ulcerative colitis, Kawasaki disease with COVID-19 vaccination in MR analyses.

Outcome	SNP (*n*)	Method	OR	*p*_value	95%(CI)
MS	51	MR Egger	4.08	0.01	1.59–10.52
MS	51	Weighted median	1.75	0.02	1.08–2.83
MS	51	Inverse variance weighted	1.53	0.02	1.06–2.20
MS	51	Simple mode	2.22	0.16	0.75–6.54
MS	51	Weighted mode	2.09	0.18	0.73–5.98
UC	28	MR Egger	1.00	0.52	0.99–1.01
UC	28	Weighted median	1.00	0.30	1.00–1.00
UC	28	Inverse variance weighted	1.00	0.04	1.00–1.00
UC	28	Simple mode	1.00	0.47	1.00–1.01
UC	28	Weighted mode	1.00	0.50	1.00–1.00
Kawasaki disease	1	Wald ratio	5982.14	0.04	1.43–25033402.50

MS, multiple sclerosis; UC, ulcerative colitis; CI, confidence interval; IVW, inverse-variance weighted; OR, odds ratio.

**Figure 4:**
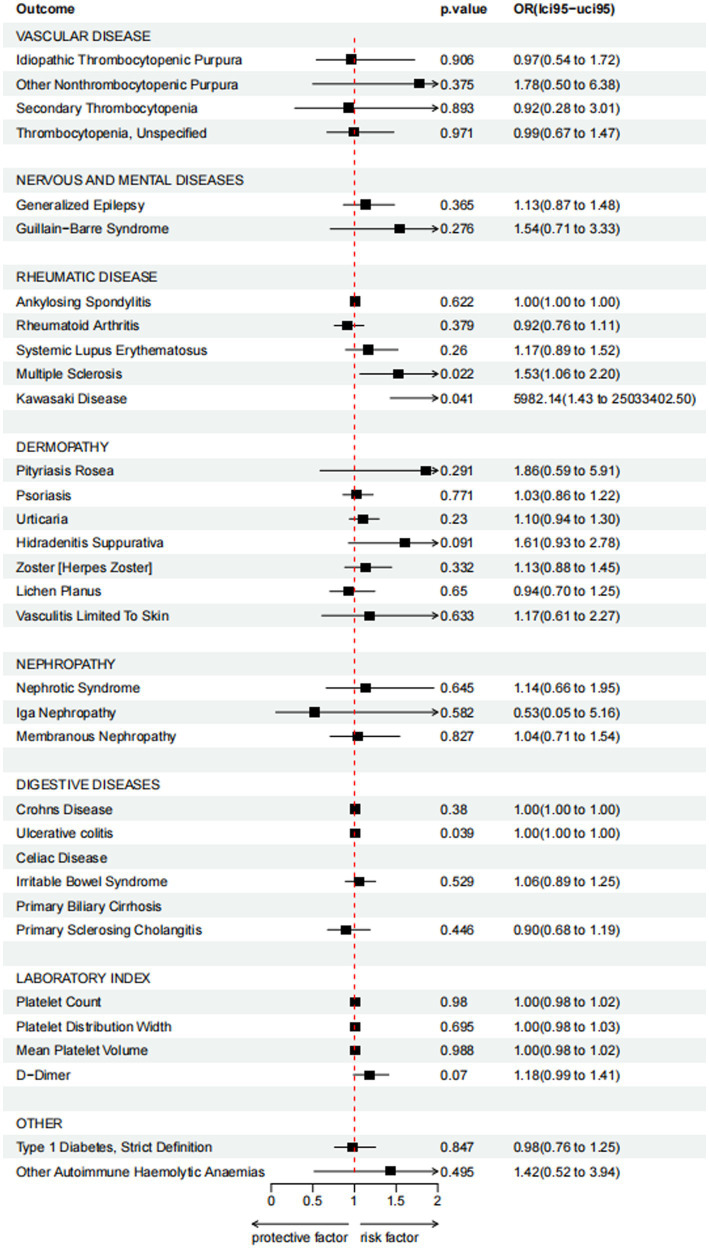
Forest plot for causal effects of COVID-19 vaccination on 31 outcomes.

Besides, as there was only one SNP for Kawasaki disease (rs4697952) after harmonization, we performed MR analysis using the Wald ratio method, which showed that vaccination had the potential to increase Kawasaki disease (Wald ratio, OR: 5982.14, 95% CI: 1.430–25033402.497, *p* = 0.041) (Table 3). Other diseases and details are in Supplementary Table S4 and Figure 4.

Then, to further validate the causal relationship between MS and COVID-19 vaccination, we have further narrowed the requirement of instrumental variables (*p* < 5 × 10^−8^; r^2^ < 0.001, genetic distance = 10,000 kb) and proceeded with MR analysis again. It was shown that there was no significant association between MS and COVID-19 vaccination with this selection of IVs (IVW, OR: 1.25, 95% CI: 0.433–3.594, *p* = 0.682), while the other 4 methods results were consistent with IVW. Likewise, UC and Kawasaki disease was no longer statistically significant with COVID-19 vaccination.

### Sensitivity analyses

In this study, Cochran’s Q test, and MR-Egger intercept test were used to further assess the robustness of the above results. However, most MR-Egger intercept tests had *p*-values larger than 0.05, indicating that there was little horizontal pleiotropy for the 31 outcomes (Supplementary Table S5). Likewise, the *p*-values for mostly Cochran’s Q tests were > 0.05, further indicating no significant heterogeneity in the results (Supplementary Table S5). Moreover, leave-one-out analysis for MS and UC revealed that no SNP drove the results, and the funnel plot was symmetrical (Figures 2A–C, 3A–C).

## Discussion

This study was conducted using MR analysis to investigate any causal effect of COVID-19 vaccination on 31 autoimmune diseases at the genetically inherited level. Our results satisfied the three assumptions of MR and indicated that COVID-19 vaccination had the potential to cause an increase in multiple sclerosis and ulcerative colitis, while the remaining findings showed no evidence of a positive or negative causality. But this positive result was based on the threshold with *p* < 5 × 10^−6^, and when we strengthened the rigour on the threshold by *p* < 5 × 10^−8^, the association between that them became negative, suggesting that the relationship between COVID-19 vaccination and MS or UC are not robust. Moreover, Kawasaki disease was substantiated as a risk factor, but it lacks robustness given that only 1 SNP was screened for harmonization.

A variety of SARS-CoV-2 vaccine types have been put into clinical service, and they are classified as live/attenuated, non-live/inactivated, and gene-based vaccines (27). According to WHO, 287 vaccines against SARS-CoV-2 are currently in development, 185 in the preclinical phase, 102 in the clinical phase, 18 in phase III, and 5 in phase IV (as of 11 June 2021, 27). Live vaccines have strong immunity and often replicate in an uncontrolled manner, which could explain their association with the risk of symptomatic disease, especially in immunocompromised individuals, and lead to some restrictions on their use (28).

Multiple sclerosis (MS) is a chronic, inflammatory, autoimmune disease for the central nervous system (CNS) characterized by the dissemination of demyelinating plaques in time and space, with pathophysiological pathways that include balance impairments caused by cerebellar, vestibular, or deep sensory dysfunction (29). The pathogenesis of MS was still uncertain, but genetic alterations, obesity, smoking, and especially, EBV infection have been implicated as etiologic factor (30, 31), which reinforced the hypothesis that viral vaccines could act as triggers for immune abnormalities leading to inflammatory demyelinating CNS disorders ultimately (32).

Human coronaviruses (HCoV) are recognized respiratory pathogens, and several strains, including HCoV-OC43, can infect human neuronal and glial cells in the central nervous system (CNS) and activate neuroinflammatory mechanisms (33). Moreover, it has been reported that as one of the coronaviruses, SARS-CoV-2 infection has been proposed to cause relapse of MS (34). Ancha Baranova et al. concluded from MR analysis that hospitalized COVID-19 increases the risk of multiple sclerosis by 15% (35). Moreover, several cases of MS have been published that may be associated with COVID-19 vaccination (36). A case report described a 40-year-old woman who developed cervical myelitis after COVID-19 vaccination, which is considered to be the initial clinical manifestation of MS (37). A 33-year-old male presented with partial right upper and lower extremity numbness 2 weeks after vaccination with Johnson & Johnson’s Janssen COVID-19 vaccine. After an MRI test and treatment, he was diagnosed with MS (38).

It is also worth noting that many neurological disorders in addition to MS have been identified after COVID-19 vaccination. A previously healthy 23-year-old male and a 33-year-old female with a previous history of depression developed neurological symptoms approximately 1 week after receiving the first COVID-19 mRNA vaccination, presenting with headache, retrograde amnesia, visual disturbances, dysarthria, tremor of the left forearm, and dullness of sensation in the mouth and distal extremities etc. Diffusion-weighted and fluid-attenuated inversion recovery MRI of the brain showed a high signal intensity lesion in the splenic midline of the corpus callosum, consistent with corpus callosum cytotoxic lesions (CLOCCs). After high-dose intravenous methylprednisolone, both patients showed significant improvement in symptoms and imaging (39). The potential role of vaccine-induced immunity is suggested by the case report of Tina Y. Poussaint et al., which resulted in MIS-C-like symptoms in a recently vaccinated child with cytotoxic lesions of the corpus callosum (CLOCC) (40). However, this case is not clearly representative because the child has a history of Lyme disease, which makes his immune function unstable.

On the other hand, GWAS in MS were concentrated in the spleen, blood, small intestine, and lungs, instead of the brain, which were the three most relevant tissues for COVID-19. Enriched tissues provide another layer of proof for MS and COVID-19 connections (35). There are five overlapping protein-coding genes between GWAS hits in COVID-19 and MS, which may contribute to the common pathophysiology of the two diseases. These include *CDC37, PDE4A*, and *KEAP1* on chromosome 19p13.2, as well as *TNFAIP8* and *HSD17B4* on chromosome 5q23.1.

However, the mechanisms underlying the CNS inflammation induced by the COVID-19 vaccine are not yet clear (35). It is well known that SARS-CoV-2 is able to cross the blood–brain barrier (BBB) and cause inflammation of the CNS (41), so many of the neurological manifestations were reported in COVID-19. Vaccines and adjuvants are known to induce autoimmunity since the structure-associated host proteins react with those in the vaccine (42). The pathogenesis of demyelinating autoimmune diseases may involve interactions between host proteins and antibodies to the COVID-19 S-protein (42). Besides, data suggested that vascular wall cells in the human brain may express low-level ACE2 (41), which could have interactions with viral S proteins and induce inflammation (42).

Furthermore, each vaccine affects a different signaling pathway. For example, the mRNA vaccine initiates the major RNA sensor TLR7, which could induce naive T cells to secrete IL-1, IL-6, and IL-12 and differentiate into Th1 and Th17 cells, releasing IL-17 and IFN-γ (43). Meanwhile, IL-6, IL-1β, tumor necrosis factor (TNF), and IL-17, which were known as SARS-CoV-2-related cytokines, disrupted the blood–brain barrier and may facilitate viral entry (44). Thus, Immune cells recognized vaccine-associated antigens and activated immune cells, which produce inflammatory cytokines, causing a cytokine storm that leads to demyelination, driving MS the relapse and triggering of MS (45).

The risk of MS as an autoimmune disease is easily triggered by immune disorders, and infectious diseases may also raise the risk of MS progression (46). However, there was no evidence of a long-term association between vaccination and demyelinating disease, although a large case–control study published in 2014 suggested that vaccination may accelerate the transition from subclinical to overt demyelination (47). A study by Hapfelmeier et al. showed that vaccination was associated with a lower likelihood of evoking MS over the next 5 years (48). Many studies have provided support for the idea that the COVID-19 vaccine is safe for patients with MS. In a study enrolling 250 patients with MS, the rate of pseudo-relapse was no higher than 5% after receiving 2 doses of BNT162b2 or mRNA-1273 (49).

As for UC, there was no evidence to suggest that COVID-19 vaccination increases the incidence of UC so far. The Partnership to Report Effectiveness of Vaccination in populations Excluded from initial Trials of Coronavirus Disease (PREVENT-COVID) evaluated the incidence of adverse events within 7 days of receiving mRNA or adenovirus-vectored vaccines in 3316 patients with IBD. The most common adverse reactions were injection site pressure or pain, and serious adverse events (defined as reactions that prevented daily activities) were rare in general. The overall incidence of IBD outbreaks after vaccination (2%) was low (50). Hyun Jeong Ju et al. included a total of 3,838,120 COVID-19-vaccinated individuals and 3,834,804 unvaccinated people for comparison, suggesting that the risk of various immune disorders, including ulcerative colitis, was not significantly higher in vaccinated individuals than in controls (51). Similarly, to explore IBD outcomes, Raffi Lev-Tzion et al. compared 707 vaccinated IBD patients with unvaccinated IBD patients by strict matching found that the risk of acute exacerbation was 29% for vaccinated patients versus 26% for unvaccinated patients (*p* = 0.3, 52).

Nevertheless, there have been numerous reports of adverse reactions following COVID-19 vaccination globally. One of the more concerned Psoriatic Arthritis (PsA) (53), pericarditis and myocarditis (10), were negative in this study, suggesting that there were no causal relationship between these diseases and COVID-19 vaccine. Therefore, our study cannot account for the emergence of these adverse effects, and more researchers are needed to further explore this issue.

The strengths of this study were as follows: First, we used randomly assigned genetic variants to determine the causal effect of COVID-19 vaccination on outcomes, meeting the three assumptions of MR, which could reduce conventional bias. Secondly, this was a summary of a large number of adverse reactions reported after COVID-19 vaccination, which was a meaningful clinical reference, especially for public health and vaccination for special groups.

However, our study has some drawbacks. Firstly, the populations we included were all European, so the results could not be applied to the entire population. Secondly, the vaccination information selected from the GWAS database did not detail the type of vaccine, which would require additional and more thorough research to provide genetic information on the different vaccines. Thirdly, the genetic information about MS did not differentiate between new-onset or relapsed MS, given that there were quite a few reports of MS patients suffering from MS relapsing after vaccination. So there is a need for further research to decipher whether there’s an unknown causal relationship between COVID-19 vaccination and different types of MS.

Our findings revealed that the COVID-19 vaccination probably raises the occurrence of multiple sclerosis and ulcerative colitis. However, this causal relationship was only based on the results obtained from a certain level of data analysis. Once we refined the exposure factor and raise the threshold, the relationship becomes non-causal. So our results only served as a reference for precaution, and cannot be enough to prove the dangers of COVID-19 vaccination.

## Conclusion

In conclusion, our study provides potential evidence of the risk effect of COVID-19 vaccination on multiple sclerosis and ulcerative colitis in European populations on a certain level. However, caution is necessary when interpreting results due to limited statistical power. More studies are needed to explore the mechanisms and biological pathways of vaccines against autoimmune diseases.

## Data availability statement

The original contributions presented in the study are included in the article/Supplementary material, further inquiries can be directed to the corresponding author.

## Author contributions

JS: Conceptualization, Writing – original draft. XH: Data curation, Formal analysis, Writing – review & editing. TC: Investigation, Methodology, Writing – original draft. YW: Project administration, Resources, Writing – review & editing. BH: Software, Supervision, Writing – review & editing. YX: Visualization, Writing – original draft. HX: Supervision, Writing – review & editing.
